# Primary pulmonary histiocytic sarcoma with CNS metastasis: a case report and molecular profiling insights

**DOI:** 10.3389/fonc.2026.1636634

**Published:** 2026-02-06

**Authors:** Kai Chen, Lei Zhang, Ruotong Wu, Jingxian Wei, Kaige Yang, Chenghua Luo, Haijun Zhang, Lin Tao, Lan Yang, Lian Meng, Weixia Nong, Jianming Hu

**Affiliations:** 1 Department of Pathology and National Health Commission(NHC) Key Laboratory of Prevention and Treatment of Central High Asia Diseases Incidence, Shihezi University School of Medicine/The First Affiliated Hospital, Shihezi University, Xinjiang, China; 2 Department of Clinical Laboratory, The First Affiliated Hospital of Shihezi University, Shihezi University, Xinjiang, China; 3 Department of Hematology, The First Affiliated Hospital of Shihezi University, Shihezi University, Xinjiang, China

**Keywords:** case report, fusion genes, histiocytic sarcoma, molecular profiling, pulmonary neoplasms

## Abstract

Histiocytic sarcoma (HS), reclassified in the WHO fifth edition as a Histiocytic/dendritic cell neoplasms, represents a rare hematopoietic malignancy with extranodal predominance and aggressive clinical behavior. This study reports the case of a 53-year-old female diagnosed with primary pulmonary HS, who presented with a 60-mm mass in the right middle lobe and later developed fatal brain metastases. Using a combination of pathology, whole-exome sequencing, and fusion gene analysis, we identified key molecular drivers of tumor development and spread. Major findings include the concurrent activation of the RAS/MAPK and PI3K/mTOR pathway activation (118 combined gene variants), TP53 biallelic inactivation, HLA locus alterations, and persistent LOC285045 fusions. Drug sensitivity profiling suggested potential responses to sunitinib and MEK inhibitors. By comparing this case with nine other reported cases of lung HS, we found that lung HS has a significantly worse survival (p=0.03) than HS at other sites. A high cell growth rate (Ki-67 >30%) and large tumor size (>50 mm) were identified as critical indicators of poor prognosis.

## Introduction

Histiocytic sarcoma (HS), reclassified in the fifth edition WHO criteria as a neoplasm of Histiocytic/dendritic cell neoplasms (1), represents a rare hematopoietic malignancy derived from monocyte/macrophage precursors (2). Characterized by an extranodal presentation and aggressive clinical course., HS typically demonstrates CD163+/CD68+/lysozyme+ immunophenotype with exclusion of other histiocytic or hematopoietic malignancies for definitive diagnosis (3, 4). Owing to its rarity, epidemiological data are limited. Reported cases indicate a broad age range (from 6 months to 89 years) and a potential male predominance (5).Primary pulmonary HS is exceptionally uncommon, with only nine cases documented to date (6–14). Although the molecular pathogenesis of HS is not fully elucidated, emerging evidence implicates alterations in the RAS/MAPK pathway (e.g., NF1, MAP2K1 mutations) and TP53 inactivation in disease progression (15, 16). Notably, secondary HS frequently associates with clonal relationships to B-cell lymphomas, evidenced by shared immunoglobulin gene rearrangements (17, 18), whereas primary HS lacks such lineage-specific markers (19). Diagnosis remains challenging due to the rarity of HS and its significant morphological overlap with other malignancies. Definitive diagnosis requires histopathological evaluation and immunophenotypic analysis: tumor cells exhibit CD163+, CD68+, lysozyme+, and CD4+ staining (20), while lacking markers of Langerhans cells (CD1a), follicular dendritic cells (CD21/CD35), myeloid cells (CD13/MPO), melanoma (HMB-45), or epithelial tumors (AE1/3, EMA) (21). A comprehensive differential diagnosis is essential and must exclude morphologically similar entities. These include: classical Hodgkin lymphoma [positive for CD15, CD30, and weakly positive PAX5, but negative for CD45 and histiocytic markers [CD163, CD68] (22)]; myeloid sarcoma [positive for CD13/CD33 but negative for CD163 (23)]; Rosai-Dorfman disease [strongly S100+, negative for CD1a and HLA-DR (24]; malignant melanoma [may express S100 and CD68 but characteristically positive for HMB-45/Melan-A and negative for CD163 (25, 26)]; follicular dendritic cell sarcoma [positive for CD21/CD23/CD35, negative for CD163 (27)]; diffuse large B-cell lymphoma [expresses B-cell markers [CD20/CD79a] (28)]; epithelioid sarcoma [positive for AE1/AE3, EMA, or CD34 (29)]; and pleomorphic rhabdomyosarcoma [expresses myogenic markers [MyoD1, myogenin] (30)]. This study investigates a 53-year-old female with primary pulmonary HS presenting as a 60-mm right middle lobe mass, The disease followed an aggressive course, resulting in fatal brain metastasis within 160 days of diagnosis. Through integrated histopathological, genomic, and fusion gene analyses, we aimed to characterize the molecular drivers of tumorigenesis and metastatic progression in this case, and to evaluate potential prognostic biomarkers. Our findings offer valuable insights into the molecular taxonomy of pulmonary HS and its therapeutic vulnerabilities, thereby contributing to a more comprehensive understanding of this aggressive malignancy.

## Case presentation

**Figure 1:**
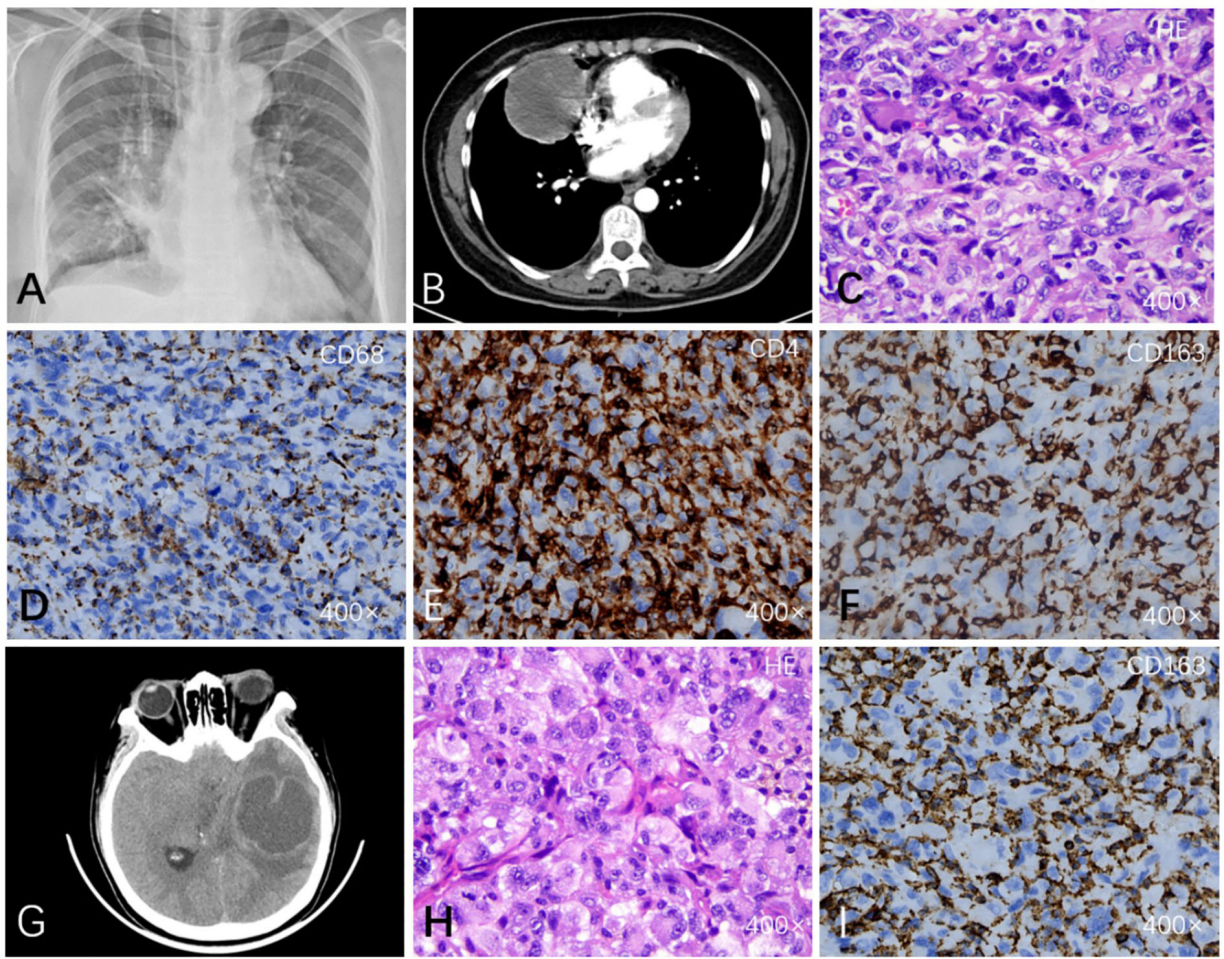
Imaging findings and histopathological microscopic observations of the tumor explanation. **(A)** Chest radiograph showing a mass in the middle lobe of the right lung. **(B)** CT scan (arterial enhancement phase) showing a 6 cm mass in the middle lobe of the right lung. **(C)** Histological examination (HE staining) reveals diffuse infiltration of tumor cells into the pulmonary parenchyma with marked cellular atypia and pleomorphism. The neoplastic cells are arranged in sheets, exhibiting hyperchromatic nuclei and prominent intercellular clefting. Multinucleated giant cells and spindle-shaped tumor cells are observed (400×). **(D)** CD68(+): a marker of macrophages and histiocytes. **(E)** Immunohistochemistry positive for CD4. **(F)** CD163(+): a specific histiocyte marker. **(G)** MRI reveals intracranial metastatic lesions. **(H, I)** Histological features (HE staining) and immunohistochemical profiles of the metastases are concordant with the primary tumor.

A woman in her 50s was admitted with a 20-day history of persistent chest pain and fever. Initial imaging at the referring hospital revealed a large right hilar mass, approximately 6 cm in diameter, associated with multiple enlarged mediastinal and cervical lymph nodes (**Figures 1A, B**). Based on these findings, a preliminary diagnosis of “probable adenocarcinoma with squamous differentiation” was made. Upon transfer to our department, laboratory tests revealed hypokalemia and mild anemia. The tumor’s location (central hilar) and imaging characteristics (massive solid mass with lymphadenopathy) were consistent with pulmonary malignancy, though the specific histological type remained unclear. Differential diagnoses included primary bronchogenic carcinoma, pulmonary lymphoma, and other rare primary or metastatic tumors. A thoracoscopic right middle lobectomy was performed for definitive diagnosis and treatment. Intraoperatively, a well-defined solid mass measuring approximately 60 × 50 × 45 mm was identified. It appeared yellowish-white with moderate consistency. The surgical margins, including the visceral pleura and bronchus, were free of tumor, and no lymph node metastasis was identified. Histopathological examination of the resected specimen (**Figure 1C**) revealed sheets of diffusely arranged tumor cells exhibiting marked pleomorphism, including giant tumor cells, and abundant mitotic figures. These morphological features were not characteristic of a typical carcinoma or a common lymphoma. To refine the diagnosis, systematic immunohistochemical analysis was performed: AE1/3, EMA, and TTF-1 were negative, strongly ruling out lung adenocarcinoma or squamous cell carcinoma; S-100 protein was negative, essentially excluding malignant melanoma; and the myogenic marker MyoD1 was negative, also excluding rhabdomyosarcoma. Key positive findings emerged for histiocyte-associated markers: Vimentin, CD163, CD68, and Lysozyme. Additionally, abnormal expression of CD56, CD4, and CD31 was observed (**Figures 1D-F**). The tumor exhibited a high proliferative index, with a Ki-67 labeling index of approximately 60%. Based on these findings, a definitive diagnosis of primary pulmonary histiocytic sarcoma was rendered. The patient remained stable during approximately 3 months of postoperative follow-up. However, on postoperative day 130, the patient presented with sudden severe headache and frequent vomiting. MRI revealed an intracranial mass lesion (**Figures 1G-I**), suspected to be metastatic, which was later pathologically confirmed as histiocytic sarcoma. Despite resection of the metastatic lesion, the patient’s condition deteriorated rapidly. This was complicated by increased intracranial pressure leading to cerebral herniation and subsequent central respiratory failure. The patient unfortunately passed away on postoperative day 160. The total disease course from symptom onset to death was approximately 180 days, highlighting the extreme aggressiveness and poor prognosis of this disease.

### Comparison and Prognosis

Primary pulmonary histiocytic sarcoma (HS) is exceedingly rare, with only nine cases reported to date (6–14) (**Table 1**). Our analysis indicates that primary pulmonary HS carries a significantly worse prognosis compared to HS arising from other extranodal sites (mean survival <1 year, p=0.03). This is notably shorter than the reported median survival range of 6 to 16 months for primary HS (regardless of site) overall (31, 32). Beyond anatomic location, disease extent and resectability are critical determinants of outcome. patients with multifocal disease have a markedly inferior median overall survival (10 months) compared to those with unifocal disease (50 months) (32). Furthermore, complete surgical resection of localized lesions (e.g., in cervical lymph nodes, stomach, or skin) can lead to long-term survival, exceeding 2, 4, or even 8 years (33–35). Against this backdrop, the dismal outcome in our patient (survival of 160 days) underscores the particularly aggressive nature of primary pulmonary HS presenting. High Ki-67 proliferation index inversely correlated with survival (correlation coefficient=-0.82, *p=0.01*). Tumor size >50 mm also predicted poor outcomes (*p=0.02*). ROC analysis identified optimal prognostic thresholds: Ki-67 >30% and tumor size >50 mm. However, limited sample size necessitates further validation.

**Table 1:** Summary of all reported histiocytic sarcoma cases occurring in pulmonary tissue.

Case No.	Age	M/F	Symptom	Tumor involvement	Therapy	Outcome	Tumor size	MIB-1 index
1	68	M	Cough, respiratory disturbance, weight loss	Lymph node (mediastinum, lung hilar, tracheal bifurcation) & lung	Chemotherapy	N.D.	68 mm	N.D.
2	3	M	Back to waist pain	Tumor in 4th vertebrae & lung	Chemo-radiation	N.D.	N.D.	N.D.
3	23	M	By chance (surgical resection of recurrent pneumothrax)	Right lung	Surgical resection	No recurrence within 1 year	4 mm	10-15%
4	16	M	By chance (surgical resection of recurrent pneumothrax)	Right lung (S6)	Surgical resection	No recurrence within 2 years	20×20×32 mm	10%
5	62	F	Chest pain and fever	Bilateral lung	N.D.	N.D.	88 mm	N.D.
6	61	M	Dyspnea, chest tightness, and productive cough with white sputum, malignant pleural effusion	Anterior mediastinal with right pleural seeding	Surgical excision, radiotherapy and chemotherapy	No recurrence within 8 months	43mm×29mm	40%~50%
7	52	M	Intermittent head motor tics, aphasia and right upper extremity weakness	Brain and lung	Surgical resection	N.D.	N.D.	N.D.
8	63	M	Acute abdominal pain	Ileum and right lung	Surgical resection	1 week	70×40mm in ileum and 130mm × 40mm in lung	N.D.
9	45	M	Cough, shortness of breath, palpitations, night sweats	Right lung lesions, mediastinal lymph nodes, right supraclavicular and iliac wings	CHOP chemotherapy (6 cycles), nivolumab/pembrolizumab immunotherapy	PR after CHOP, relapse, 3+ years PFS with immunotherapy	141 × 77 mm (initial CT), reduced to 70 × 8 mm	50%
10	53	F	Chest pain and fever	Right lung (middle lobe)	Surgical resection	160days	60 mm	60%

No.1–9 correspond to previously published reports [6-14]; No.10 represents the current case.

### Somatic SNV/indel analysis

SNV and Indel detection employ the mutect2 toolkit within the GATK software package. Preprocessing involves utilizing Picard for deduplication (Mark Duplicates) and base quality recalibration based on the alignment outcomes of Clean Reads against the reference genome. This safeguards the accuracy of detecting somatic SNVs and Indels. The primary tumor harbored 48 somatic variants in the RAS/MAPK pathway (**Supplementary Table S1**), including Indels (*RASA1, PTPN11, MAPK3, CACNA1, CDH2*) and SNVs (*HGF, PTPN11, FLT1, MAP2K1, NF1*). Additionally, 70 variants were identified in the PI3K/mTOR pathway (**Supplementary Table S2**), involving Indels (*COL9, FGFR4, HGF, JAK3, SGK2*) and SNVs (*COL9, FOXO3, MYB, PIK3R6, JAK3*). Other notable findings included epigenetic dysregulation (*EZH2* and *ATRX* SNVs), *TP53* mutations (Indel and SNV), and HLA-related SNVs (*HLA-G, HLA-E, HLA-DRA, HLA-DQA, HLA-DPB1*). Key negative results included absence of *KRAS* mutations, *CDKN2A* copy number loss, *MDM2/CDK4* amplifications, and lymphoma-associated mutations (*MYD88, CD79B, BCL6, L265P*).

### CNV/LOH analysis

**Figure 2:**
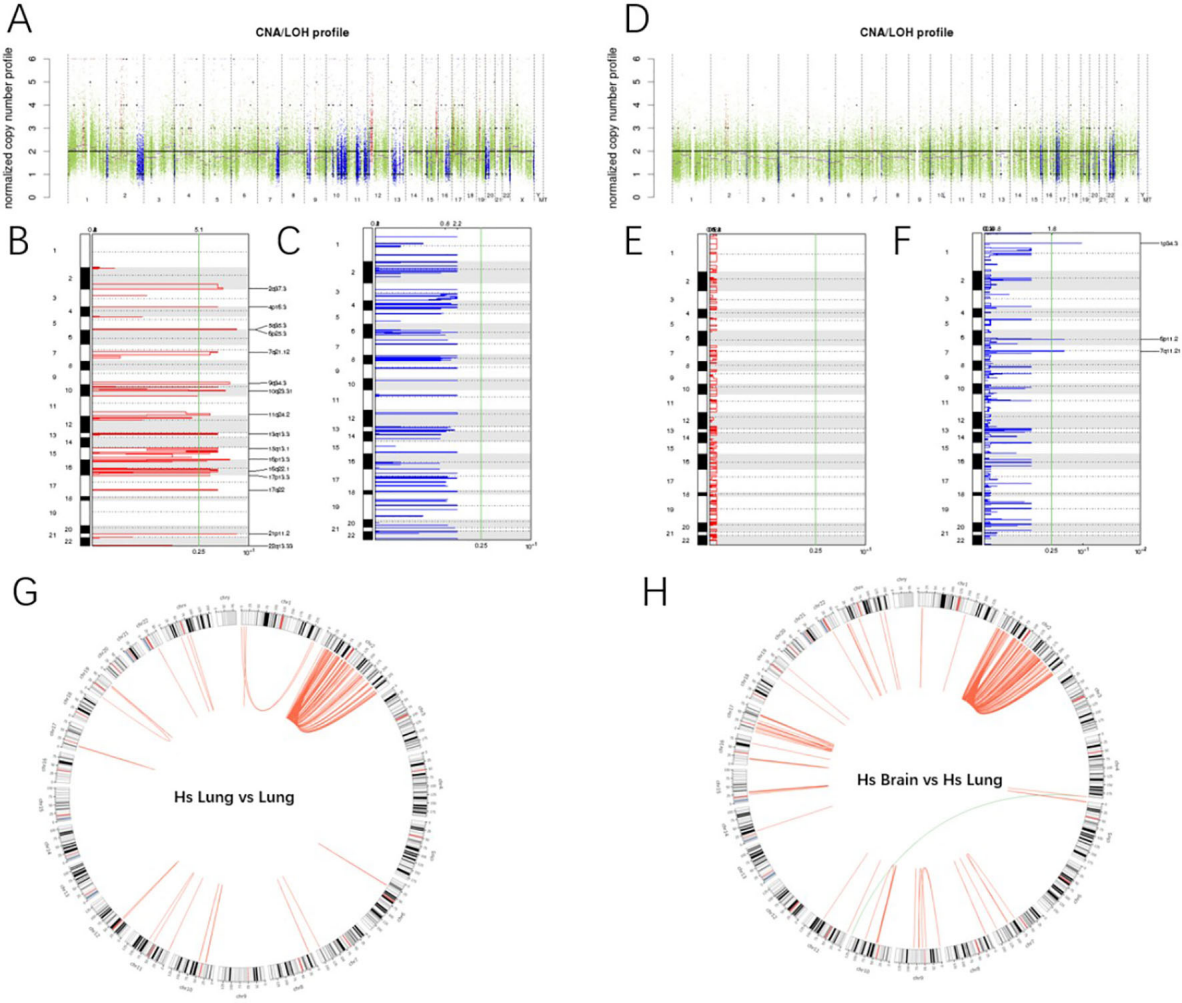
Analysis of CNV/LOH and fusion genes. **(A)** comparison of CNV and LOH between primary lung lesion and adjacent normal tissue. The horizontal axis represents distinct chromosomes. In the upper panel, red denotes copy number gain, blue signifies copy number loss, and green indicates no detectable change. **(B)** Copy Number Variations in Primary Lung Lesion Compared to Adjacent Normal Tissue. The left vertical axis denotes chromosome numbers, and the right vertical axis indicates chromosomal positions of CNV. The lower horizontal axis represents significance levels (-log_10_[q-value]), while the upper horizontal axis displays G-scores of variations (incorporating magnitude and inter-sample frequency). The green line marks the significance threshold (q-value = 0.25). **(C)** Copy Number Loss in Primary Lung Lesion Compared to Adjacent Normal Tissue. **(D-F)** Comparison of CNV and LOH between Metastatic Lesions and Primary Lung Lesion. **(G)** Fusion Genes in Primary Lung Lesion Compared to Adjacent Normal Tissue. Red lines represent intrachromosomal fusion events (occurring within the same chromosome), while green lines denote interchromosomal fusion events (occurring between distinct chromosomes). **(H)** Fusion Genes in Metastatic Lesion Compared to Primary Lung Lesion.

To detect the B Allele Frequency (BAF) at Somatic CNV and SNV loci, Control-FREEC (Boeva V et al., 2011) software is utilized. The primary tumor exhibited recurrent amplifications in 16 chromosomal regions, including 7q21.12 (encompassing *CDK6*), 22q13.33, 2q37.3, 6p25.3, 17p13.3 (containing *TP53*), 21p11.2, 16p13.3, 10q23.31, 9q34.3 (spanning *NOTCH1*), 4p16.3, 13q13.3, 5q35.3, 17q22, 15q13.1, 11q24.2, and 16q22.1 (G-score >5.1, q-value <0.25), with no significant deletions detected (**Figures 2A-C**). In contrast, the brain metastasis demonstrated deletions in 6p11.2 (HLA region), 1p34.3 (harboring *MYCL*), and 7q11.21 (including *ELN*), but lacked prominent amplifications compared to the primary pulmonary lesion (**Figures 2D-F**).

### Driver genes and predisposition

The detection of germline mutations (SNV, InDel) in a patient’s normal tissue using the Haplotype Caller tool of the GATK software is followed by a comparison of the detected mutated genes with the CGC (Cancer Gene Census) database. OncodriveCLUST analysis identified *NBPF10* as a driver gene in the primary tumor (36), characterized by six non-synonymous mutations clustered within three functional domains (*P* < 0.05, *Q* < 0.05), meeting the criteria for driver gene classification (**Supplementary Table S3**). In contrast, no significant driver genes were detected in the metastasis compared to the primary lesion. Additionally, five potential Pan-cancer cancer predisposition genes were identified in the primary tumor: *MUC6* (12 pathogenic loci), *FAT1* (2 loci), *KDR* (2 loci), *BARD1* (1 locus), and *MPL* (1 locus) (**Supplementary Table S4**).

### Fusion genes

The detection of fusion genes in tumor exomes is primarily conducted using FusionMap software. Gene fusion identification is performed separately for tumor samples and normal samples, with fusion genes that also appear in the paired normal samples from tumor samples being excluded. The primary tumor demonstrated high-frequency *LOC285045* fusions (122 events), with partners including *PRKCE*, *WNT6*, and *HOXD* family genes. Other notable fusions included *HBB-HBD* and *FTH1-RSF1* (**Figure 2G**). Metastatic lesions exhibited persistent *LOC285045* fusions (168 events) alongside novel events such as *USP6-TBC1D3*, *CT45/CT47*, and *GOLGA8* family fusions (**Figure 2H**, **Supplementary Tables S5**, **S6**).

### Drug sensitivity

IMPACT (Integrate Molecular Profiles with Actionable Therapeutics) is used to predict treatment outcomes in clinical studies. By mining the National Cancer Institute (NCI)-Match, MD Anderson Personalized Cancer Therapy (PCT), and DSigDB databases, it matches mutated genes with FDA (Food and Drug Administration) approved targeted drugs. Comprehensively compare multiple databases, among which in the group of “DSigDB FDA Approved Kinase Inhibitors” identified four FDA-approved targeted therapies: Sunitinib (FLT1 inhibitor), Pazopanib, Vandetanib, and Axitinib, supported by *FLT1* SNVs in the primary tumor. Subsequently, the database was expanded to search for more potential therapeutic targets, and combined with National Cancer Institute (NCI)-Match and MD Anderson Personalized Cancer Therapy (PCT), the potential target prediction analysis was conducted, Analysis of the overall genetic variation profile suggested potential efficacy for conventional chemotherapeutic agents. Dactinomycin(p = 0) and methotrexate(p = 0.004) showed the strongest in-silico signals, linked to 24 and 31 potential targets, respectively. Doxorubicin Hydrochloride was also significantly associated (p = 0.014) and has 104 potential targets. The activation of the RAS/MAPK pathway additionally indicated that Sorafenib tosylate might have potential therapeutic effects (p = 0.005, 42 potential targets) (**Table 2**). No biomarkers predictive of immune checkpoint inhibitor response were detected.

**Table 2:** Actionable therapeutics.

Drug	Potential gene targets(6): APEX1 CHD2 FLT1 GJA8 IGHMBP2 TP53			
	Targets hit	Potential targets	P-value (hypergeometric test)	P-value (Permutation test)
DACTINOMYCIN	2	24	0	0
methotrexate	2	31	0	0.004
Sorafenib tosylate	2	42	0	0.005
Axitinib	2	105	0.001	0.008
Irinotecan hydrochloride	1	5	0	0.011
Doxorubicin Hydrochloride	2	104	0.001	0.014
Decitabine	1	7	0	0.015
thiotepa	1	9	0	0.016
chlorambucil	1	9	0	0.016
-Fluorouracil	1	8	0	0.019
Sunitinib malate	1	9	0	0.02
carmustine	1	9	0	0.022
Clofarabine	1	12	0	0.024
GEMCITABINE HYDROCHLORIDE	1	11	0	0.027
mitomycin C	1	16	0	0.032
Trifluridine	1	14	0	0.032
Bexarotene	1	13	0	0.033

## Discussion

Histiocytic Sarcoma (HS) is an extremely rare and aggressive malignancy, as underscored by this case of a 53-year-old female with rapid central nervous system metastasis and a fatal outcome. Our case of primary pulmonary HS, along with the review of nine previously reported cases, reveals a critical contradiction: despite its classification within the typically more indolent primary HS subgroup (18, 37),. HS originating in the lungs exhibits exceptionally aggressive behavior and poor prognosis.

Owing to the scarcity of cases, no specific or universal molecular alterations have been definitively established for histiocytic sarcoma (15). Common alterations include BRAF V600E mutations, RAS/RAF/MEK/ERK pathway activation, TP53 mutations, MYC amplification, and CDKN2A deletion (15, 16). Monoclonal IgH rearrangements are frequent in secondary HS, while primary HS predominantly involves RAS/MAPK pathway mutations, notably NF1 and MAP2K1 alterations (15). Atypical molecular features have also been documented. For instance, Montalvo et al. reported a case with microsatellite stability and a low tumor mutational burden (2 mut/Mb) harboring mutations in FLT3, NOTCH2, and KMT2A (38). Other sporadic alterations include ATM, LRRK2, MYO18A, SETBP1, NOD1, TSC1, NTRK1, CBL, NOTCH1, and KMT2 mutations of uncertain significance (38). In the present case, genomic profiling revealed co-activation of the RAS/MAPK pathway (48 variants) and the PI3K/mTOR pathway (70 variants). Notably, RASA1, MAP2K1, and NF1 mutations partially overlapped with prior HS reports (15). We review previous studies, molecular profiling of 16 secondary HS cases associated with lymphoid malignancies revealed RAS/MAPK pathway gene mutations in 14/16 cases, including KRAS, BRAF, NRAS, MAP2K1, and NF1, indicating that FL-associated secondary HS frequently share a similar mutational profile (39). Furthermore, a comprehensive genomic study of 21 primary HS cases utilizing whole-exome and RNA sequencing identified alterations in RAS/RAF/MAPK pathway genes in all cases (100%), including NF1, MAP2K1, PTPN11, BRAF, KRAS, NRAS, and LZTR1 (40). This study further classified cases into two molecular subgroups based on gene expression: one associated with NF1/PTPN11 abnormalities, and the other linked to B-cell lymphoma-associated gene mutations and clonal immunoglobulin gene rearrangements. This conclusion and the classification basis were further confirmed in this case of primary lung HS. In our case, biallelic inactivation of TP53 (via indel/SNV) and single nucleotide variants in HLA loci (HLA-G, HLA-E, HLA-DRA) were identified, which may contribute to genomic instability and immune evasion. Given the rarity of this case, we have made detailed records of it. Although its significance is still at an initial stage, this detailed record is intended to provide a potential clue for future collaborative research.

Copy number amplifications at 17p13.3 (TP53) and 7q21.12 (CDK6) in the primary lesion correlated with a high Ki-67 index (60%), reflecting uncontrolled proliferation. The recurrent LOC285045 fusion in both primary (122 reads) and metastatic lesions (168 reads) suggests a core oncogenic role. While its precise mechanistic role requires functional validation, its consistent presence underscores its potential importance in both tumorigenesis and metastatic progression. Notably, the brain metastasis exhibited focal deletions absent in the primary tumor. HLA-I is frequently lost in various types of cancer, including colorectal cancer (CRC), leading to tumor immune escape by cytotoxic T lymphocytes during the natural history of cancer development (41). Although there is insufficient evidence to show that the MYCL genotype is associated with tumor progression or poorer prognosis, LOH at MYCL is significantly correlated with regional lymph node metastasis and advanced TNM stage (42). ELN is a core component of elastic fibers, and it is beneficial for extracellular matrix integrity. Its absence may have facilitated the infiltration of the central nervous system (43). Although these specificities were previously not reported in HS, their simultaneous occurrence during metastasis may suggest that they are crucial for successful metastatic colonization. The USP6-TBC1D3 fusion may promote invasion via RAS signaling or cytoskeletal remodeling. TBC1D3, as a tumor-specific oncogene, can enhance the activation of RAS (44). Furthermore, USP6 evolved through the fusion of the TBC1D3 and USP32 genes. Therefore, the USP6 protein retains the homologous domain of TBC1D3 (45). Therefore, we hypothesize that the novel USP6-TBC1D3 fusion identified in the metastatic lesion may have contributed to the aggressive phenotype, potentially facilitating central nervous system metastasis through mechanisms such as enhanced RAS signaling or cytoskeletal remodeling. Functional studies are warranted to validate this potential role.

Pharmacogenomic analysis identified actionable targets (e.g., FLT1 SNVs supporting sunitinib; Activation of the RAS/MAPK pathway indicates sorafenib sulfonate), though the absence of immune checkpoint markers limits immunotherapy options (27). Recent advances in molecular subtyping of HS have opened new avenues for targeted therapeutic strategies. HS cases harboring activating mutations in genes such as MAP2K1 or PTPN11 have demonstrated sensitivity to MEK inhibitors (e.g., trametinib) (46), in secondary HS, the combination of dabrafenib targeting the BRAF V600E mutation with trametinib has successfully induced sustained remission (47). Additionally, therapies targeting other molecules are increasingly mature. For instance, in patients harboring PTEN mutations, the mTOR inhibitor sirolimus serves as an effective treatment option (48). The functional significance of the LOC285045 variant identified here remains uncertain, highlighting the need for functional validation and investigation in larger cohorts.

Given the rarity of HS, no standardized treatment protocol has been established. Complete surgical resection remains the cornerstone of management for localized disease. Regarding targeted therapies, agents such as sunitinib (an FLT1 inhibitor), pazopanib, vandetanib, and axitinib may hold therapeutic potential. Analysis using DSigDB linked potential platinum sensitivity to mutations in APEX1, CHD2, and TP53. The presence of activating alterations in MAP2K1 and NTRK1 suggests potential efficacy of MEK inhibitors (e.g., trametinib) or TRK inhibitors (e.g., larotrectinib), respectively. For advanced disease, the CHOP regimen (cyclophosphamide, doxorubicin, vincristine, prednisone) is commonly employed (49), although a definitive survival benefit has not been conclusively demonstrated (50). PD-L1 overexpression was reported in 3 of 12 cases by Facchetti et al., suggesting a potential role for immunotherapy in a subset of patients (27), however, supporting evidence remains limited. Postoperative adjuvant therapy and chemotherapy remain controversial due to HS’s high-grade malignancy and cellular maturity. Current therapeutic insights, which are largely derived from case reports and small series, necessitate validation through larger, collaborative studies.

## Data Availability

The human genomic sequencing data generated during this study are not publicly available due to patient privacy and ethical restrictions. De-identified, derived data supporting the findings of this study are available from the corresponding author upon reasonable request for the purpose of validating the results. Any data sharing will be subject to the approval of the relevant ethics committee and the execution of a formal data use agreement.
